# BMP Receptor Inhibition Enhances Tissue Repair in Endoglin Heterozygous Mice

**DOI:** 10.3390/ijms22042010

**Published:** 2021-02-18

**Authors:** Wineke Bakker, Calinda K. E. Dingenouts, Kirsten Lodder, Karien C. Wiesmeijer, Alwin de Jong, Kondababu Kurakula, Hans-Jurgen J. Mager, Anke M. Smits, Margreet R. de Vries, Paul H. A. Quax, Marie José T. H. Goumans

**Affiliations:** 1 Department of Cell and Chemical Biology, Leiden University Medical Center, 2333 ZC Leiden, The Netherlands; wineke.bakker@gmail.com (W.B.); Calinda.Dingenouts@gmail.com (C.K.E.D.); k.lodder@lumc.nl (K.L.); c.c.wiesmeijer@lumc.nl (K.C.W.); K.B.Kurakula@lumc.nl (K.K.); a.m.smits@lumc.nl (A.M.S.); 2 Department of Surgery, Leiden University Medical Center, 2333 ZC Leiden, The Netherlands; A.de_Jong.HLK@lumc.nl (A.d.J.); m.r.de_vries@lumc.nl (M.R.d.V.); P.H.A.Quax@lumc.nl (P.H.A.Q.); 3 St. Antonius Hospital, 3435 CM Nieuwegein, The Netherlands; jmager@antoniusziekenhuis.nl

**Keywords:** transforming growth factor-β, endoglin, neovascularization, tissue repair, myocardial infarction, hind limb ischemia

## Abstract

Hereditary hemorrhagic telangiectasia type 1 (HHT1) is a severe vascular disorder caused by mutations in the TGFβ/BMP co-receptor *endoglin*. Endoglin haploinsufficiency results in vascular malformations and impaired neoangiogenesis. Furthermore, HHT1 patients display an impaired immune response. To date it is not fully understood how *endoglin* haploinsufficient immune cells contribute to HHT1 pathology. Therefore, we investigated the immune response during tissue repair in *Eng+/−* mice, a model for HHT1. *Eng*+/− mice exhibited prolonged infiltration of macrophages after experimentally induced myocardial infarction. Moreover, there was an increased number of inflammatory M1-like macrophages (Ly6C^high^/CD206^−^) at the expense of reparative M2-like macrophages (Ly6C^low^/CD206^+^). Interestingly, HHT1 patients also showed an increased number of inflammatory macrophages. In vitro analysis revealed that TGFβ-induced differentiation of *Eng+/−* monocytes into M2-like macrophages was blunted. Inhibiting BMP signaling by treating monocytes with LDN-193189 normalized their differentiation. Finally, LDN treatment improved heart function after MI and enhanced vascularization in both wild type and *Eng+/−* mice. The beneficial effect of LDN was also observed in the hind limb ischemia model. While blood flow recovery was hampered in vehicle-treated animals, LDN treatment improved tissue perfusion recovery in *Eng+/−* mice. In conclusion, BMPR kinase inhibition restored HHT1 macrophage imbalance in vitro and improved tissue repair after ischemic injury in *Eng+/−* mice.

## 1. Introduction

Endoglin (also known as CD105) is a transmembrane protein that functions as a co-receptor for transforming growth factor-β (TGFβ)1 and TGFβ3. Mutations in *endoglin* resulting in haploinsufficiency are the cause of the autosomal dominant vascular disorder hereditary hemorrhagic telangiectasia type 1 (HHT1). HHT1 is rare life-threatening disorder characterized by local angiodysplasia like arterial venous malformations, telangiectasia, and recurrent epistaxis. Besides vascular dysplasia, an impaired immune response was also observed in HHT1 patients, evident by e.g., increased infection rates in the brain, joints, and liver [1]. To gain more insight into the etiology of HHT1, the murine model for HHT1, the *endoglin* heterozygous (*Eng+/−*) mouse, was extensively studied. Similar to HHT1 patients, *Eng+/−* mice display decreased wound healing [2] and impaired resolution of inflammation [3]. We previously showed that *Eng+/−* mice also have a delay in blood flow recovery and a reduction of collateral artery and capillary formation after hind limb ischemia (HLI) [4]. Furthermore, *Eng+/−* reduced myocardial repair after experimentally induced myocardial infarction (MI) [5], and systemic application of wild type mononuclear cells (MNCs) stimulated revascularization of the injured myocardium and restored cardiac recovery of *Eng+/−* mice, an effect not seen when MNCs of HHT1 patients were used [5]. The exact role of endoglin in inflammation and tissue repair was not yet completely understood, but a rapid increase in expression levels of endoglin during the inflammatory phase of wound healing suggests endoglin was involved in these processes [6,7,8]. Furthermore, while in healthy individuals the expression of endoglin was upregulated in activated monocytes [9], this response was impaired both in HHT1 patients [10] and *Eng+/−* mice [11], resulting in an increased infection rate and leukopenia [1,3,11,12], for example.

Endoglin exerts its effect by modulating TGFβ and bone morphogenetic protein (BMP) signaling, two pathways proven to be essential during cardiovascular development and disease [13,14], inflammation, and tissue repair [15,16,17,18,19,20]. TGFβ is the prototypic member of a large family of growth factors to which the activins and BMPs belong [13]. Upon tissue damage, TGFβ is released from the extracellular matrix, or is secreted by activated fibroblasts, endothelial cells, platelets, and macrophages [21,22,23]. TGFβ signaling is initiated by binding of the ligand to the TGFβ type II (TβRII) transmembrane receptor. In endothelial cells and macrophages, TGFβ can propagate the signal by forming a complex between the TβRII and a type I receptor, also known as activin receptor-like kinase (ALK). Signaling via the type I receptor ALK5 results in phosphorylation of the transcription factors Small mothers against decapentaplegic (SMAD)2 and SMAD3. Complex formation of TβRII with the type I receptor ALK1 is followed by activation of SMAD1 and SMAD5. ALK1 can only signal via TGFβ by forming a heterotetrameric complex consisting of two TβRII receptors, ALK1 and ALK5, in the presence of the co-receptor endoglin damping the TGFβ/ALK5 signaling pathway [13,24,25]. In the absence of endoglin, major vascular defects and impaired angiogenesis are observed, which can only partly be explained by malfunctioning of the endothelial cells by enhanced TGFβ/ALK5 signaling. It is, however, not known what the role of endoglin deficiency in monocytes entails and how this contributes to vascular repair after an ischemic event.

Cardiac wound healing after ischemic injury can be divided in three phases—the ischemic phase, the inflammatory phase, and the repair phase [26]. During the ischemic phase, cells within the obstructed area are devoid of oxygen and nutrients and go into apoptosis or necrosis. In the inflammatory phase, cellular debris within the injured myocardium is resolved by the recruitment of immune cells, inflammatory-like macrophages (M1-like, from here onwards referred to as ‘M1’), the secretion of cytokines and degradation of extracellular matrix [26]. Approximately 5 days post-MI, the repair phase starts which is hallmarked by the release of growth factors and cytokines stimulating vascularization, recruitment of endothelial progenitor cells, and differentiation of reparative/regenerative-like macrophages (M2-like, from here onwards referred to as ‘M2’) [26,27]. The immune cells resolve after 2–3 weeks and a fibrous scar is formed [26]. Although we earlier showed that there is an impaired vascular recovery after ischemic injury in *Eng+/−* mice using two different models [4,5], as well as an imbalance in M1/M2 macrophages [28], the relation between these two observations and *endoglin* heterozygosity is still poorly understood. Therefore, the aim of this study was to elucidate the effect of *endoglin* heterozygosity on M1 and M2 macrophages during the different phases of cardiac wound healing and vascular recovery. We showed that the differentiation of monocytes isolated from the *Eng+/−* mouse into M2 macrophages contributed to the impaired tissue repair. Moreover, the impaired macrophage differentiation was confirmed in monocytes of HHT1 patients, which could be restored in vitro by inhibiting BMP signaling. Finally, BMP receptor kinase inhibition improved tissue repair of both the *Eng+/−* ischemic myocardium as well as the *Eng+/−* ischemic hind limb, by increasing neovascularization.

## 2. Results

### 2.1. Endoglin Deficiency Results in Prolonged Inflammation and Reduced M2 Macrophage Presence in the Heart after MI

Recruitment of inflammatory cells and their timely resolution is essential for cardiac tissue repair. An inadequate or excessive inflammatory response is detrimental in injured myocardium and can lead to adverse remodeling. We therefore investigated the effects of *endoglin* heterozygosity on the influx of monocytes during the inflammation phase, after experimentally inducing MI. We first determined if *endoglin* heterozygosity influences MNC composition at baseline. Before induction of MI, we observed no differences in MNC subtypes, like macrophages, lymphocytes, NK-cells, neutrophils, and granulocytes in blood and bone marrow between wild type (WT, *Eng+/+*) and *Eng+/−* mice (data not shown).

**Figure 1:**
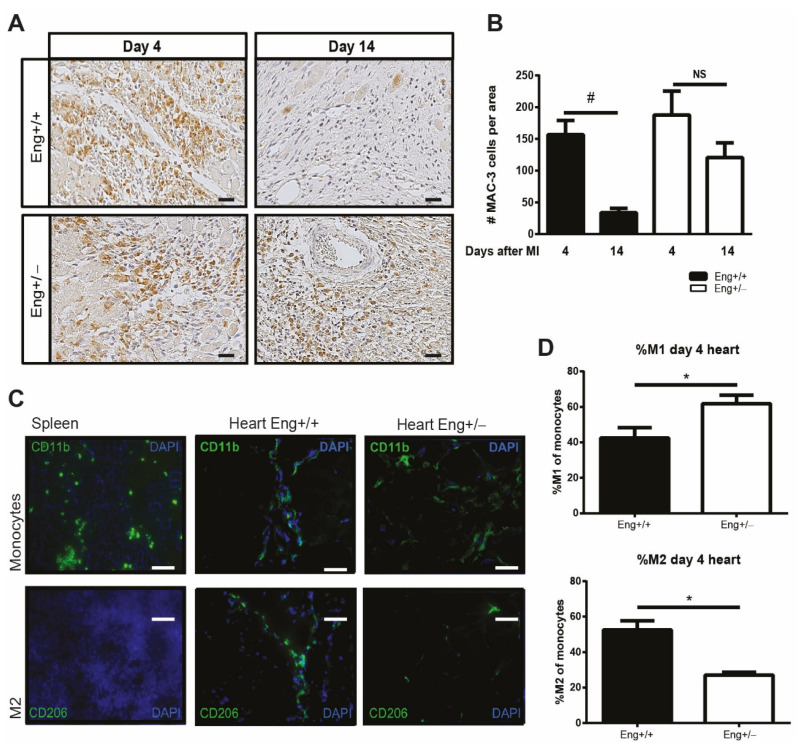
Prolonged macrophage infiltration and decreased number of M2 macrophages after myocardial infarction in *Eng+/−*. (**A**) Cardiac sections of *Eng+/+* and *Eng+/−* mice were stained for MAC-3 expressing macrophage (MAC-3 = brown; nuclei = blue) at day 4 and 14 post MI. Scale bar: 50 μm. (**B**) Quantification of the MAC3 positive cells shown in an N = 5–16 mice per group. (**C**) Splenic and cardiac tissue post-MI were stained for CD11b (general monocyte marker) and CD206 (M2 macrophage marker) 4 days after MI. Scale bar: 50 μm. (**D**) The ratio M1/M2 macrophages was determined by flow cytometry using a single cell suspension of Eng+/+ and Eng+/− mouse hearts 4 days post MI. The inflammatory M1 macrophage was identified by CD11b+/Ly6Chigh/CD206- selection and the regenerative M2 by CD11b+/Ly6Clow/CD206+ selection. N = 5–16 mice per group. *Eng+/+* N = 9, *Eng+/−* N = 5. * *p* < 0.05, # *p* < 0.001, NS: not significant.

Subsequently we induced MI and assessed the number of macrophages present in the heart using immunohistochemical analysis. Four days post-MI, MAC-3 expressing macrophages were present in large numbers in the border zone of the infarcted hearts of both WT and *Eng+/−* mice (Figure 1A,B). Macrophage infiltration in WT hearts was cleared 14 days post MI (Figure 1A,B), confirming previous studies that reported that the inflammatory response is most pronounced at day 3–5 and cleared after approximately two weeks [26]. Interestingly, at 14 days post-MI MAC-3 expressing cells were still easily detectable in the infarct border zone of *Eng+/−* mice (Figure 1B), suggesting a delay in macrophage resolution.

To further characterize the phenotype of these macrophages, we determined the expression of CD11b, a general monocyte/macrophage marker, and CD206, a specific marker for M2 (reparative) macrophages facilitating the healing process. Immunofluorescent analysis of the spleen, used as control tissue, showed the presence of CD11b-positive resident monocytes, while no CD206 staining was observed (Figure 1C). Four days post-MI, hearts of WT mice harbored similar numbers of CD11b and CD206 expressing cells, suggesting that the macrophages present in the heart were M2 macrophages. In contrast, in the hearts of *Eng+/−* mice, CD11b+ cells were easily detectable, while only limited numbers of CD206 expressing cells were present. We quantified these observations using flow cytometry on single cell suspensions of mouse hearts (Figure 1D). At day 4 post-MI, the *Eng+/−* hearts contained significantly less M2 macrophages, while the number of pro-inflammatory M1 macrophages was significantly increased (Figure 1D). This suggests a macrophage polarization in the injured *Eng+/−* heart towards a more inflammatory macrophage phenotype.

### 2.2. Endoglin Deficiency Reduces In Vitro Differentiation of M2 Macrophages in Both HHT1 Mice and Patients

**Figure 2:**
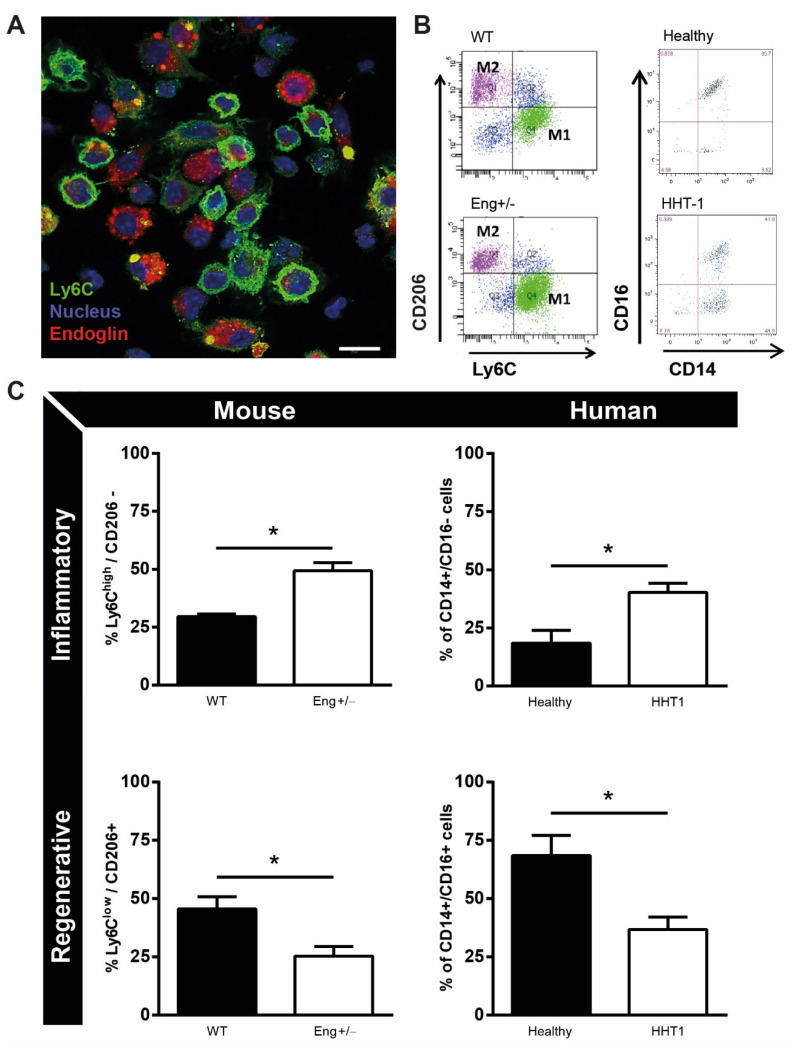
Macrophage phenotype is dependent on endoglin expression. (**A**) Macrophages isolated from *Eng+/+* mice were stained with endoglin (red), Ly6C (green), and dapi (nuclei, blue). Scale bar: 10μm. (**B**) Representative flow charts of mouse and human isolated monocytes of *Eng+/−* mice and HHT1 patients and their healthy controls. Mouse inflammatory monocytes were distinguished by CD11b+/Ly6Chigh/CD206- and regenerative monocytes by CD11b+/Ly6Clow/CD206+ expression. Human inflammatory monocytes were distinguished by CD14+CD16- and regenerative monocytes by CD14+/CD16+ expression. (**C**) Quantification of the flow cytometry data as represented in B, divided in inflammatory and regenerative monocytes for mouse and human. Mouse samples: N = 5–16 mice per group. *Eng+/+* N = 9, *Eng+/−* N = 5. Human samples: 7 HHT1 patients and 5 age- and gender-matched healthy human volunteers. * *p* < 0.05.

To gain more insight into how *endoglin* heterozygosity might influence macrophage differentiation, we isolated bone marrow derived CD11b+ monocytes and used immunofluorescence to analyze their differentiation towards macrophages in vitro. Endoglin is expressed on murine macrophages and co-staining of endoglin together with the inflammatory macrophage marker Ly6C, revealed that endoglin is specifically present on murine macrophages with a low expression level of Ly6C (Figure 2A).

Macrophages with high expression levels of Ly6C, known as the inflammatory-like (M1-like) subtype, show low expression levels of endoglin (Figure 2A). More detailed analysis of the different macrophage subtypes in *Eng+/−* mice and HHT1 patients was performed using flow cytometry. Inflammatory (M1) macrophages were identified by the expression of CD11b, high levels of Ly6C and low CD206 expression for mouse macrophages, and the expression of CD14 and absence of CD16 expression for human cells (Figure 2B).

Reparative (M2) macrophages were identified by the expression of CD11b, low levels of Ly6C and high expression of CD206 for mouse, and the expression of both CD14 and CD16 for human cells (Figure 2B). Monocytes isolated from *Eng+/−* mice as well as HHT1 patients showed an increased percentage of inflammatory macrophages and a reduction of reparative macrophages, compared to macrophages from WT mice and healthy volunteers (Figure 2C).

Reparative (M2) macrophages were identified by the expression of CD11b, low levels of Ly6C and high expression of CD206 for mice, and the expression of both CD14 and CD16 for human cells (Figure 2B). Monocytes isolated from *endoglin* heterozygous mice as well as HHT1 patients showed an increased percentage of inflammatory macrophages and a reduction of reparative macrophages, compared to macrophages from wildtype mice and healthy volunteers (Figure 2C). Interestingly, monocytes from endoglin heterozygous mice secrete more MCP-1 compared to wild-type cells, confirming their increased inflammatory profile (Supplementary Figure S1).

### 2.3. In Vitro Switch of Macrophage Differentiation by Adaptation of the TGFβ Signaling Response

**Figure 3:**
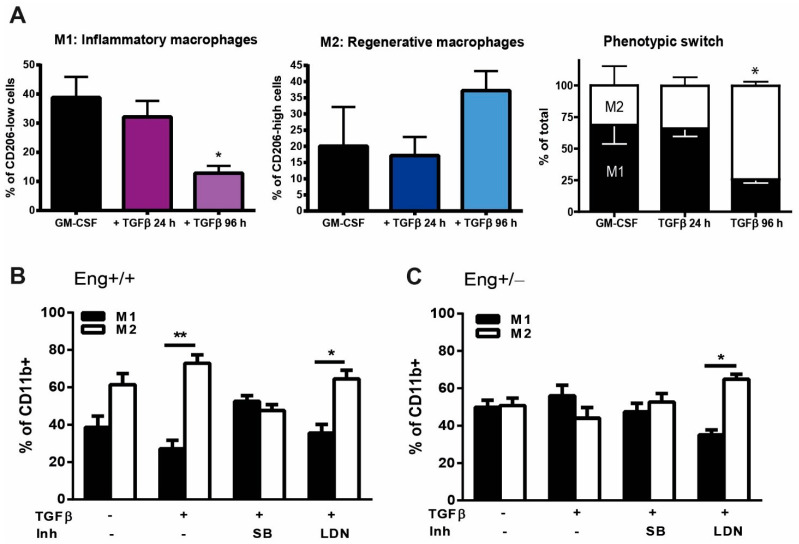
TGFβ signaling influences macrophage subtype differentiation. (**A**) Macrophages from *Eng+/+* mice cultured with either GM-CSF for 7 days or in the presence of TGFβ (2.5 ng/µL) for 24 h and 96 h. The macrophage phenotype was determined based on the expression of Ly6C high (M1) and low (M2) of the CD11b expressing macrophages. * *p* = 0.001 difference in the number of M1 and M2 between GM-CSF vs. TGFβ stimulation for 96 h. (**B**,**C**) BM isolated monocytes from *Eng+/+* (**B**) and *Eng+/−* (**C**) mice were cultured in the presence of GM-CSF in the presence or absence of TGFβ (2.5 ng/µL), SB (10 μM), or LDN (100 nM). The macrophage subtype was determined based on the expression of Ly6C high (M1) and low (M2) of the CD11b expressing macrophages. * *p* = 0.007; ** *p* < 0.0001.

As endoglin is a co-receptor for the TGFβ signaling cascade, we next investigated the effect of stimulation and inhibition of the TGFβ-signaling pathway on macrophage differentiation. Monocytes isolated from bone marrow of WT mice were cultured for 3 days in the presence of GM-CSF to stimulate their differentiation into macrophages, after which 1ng/mL of TGFβ ligand was added for either 24 or 96 h. While 24 h of TGFβ stimulation had little effect on the percentage of M1 and M2 macrophages compared to the non-stimulated cells, 96 h of TGFβ stimulation skewed macrophage differentiation towards an M2 phenotype (Figure 3A).

This TGFβ increase in M2 macrophages was blocked by adding SB-431542 (SB), a potent ALK5 kinase inhibitor, to monocyte cultures from wildtype mice, but was not affected by adding the BMPRI kinase inhibitor LDN-193189 (LDN) (Figure 3B). The M1/M2 macrophage numbers did not change when monocytes isolated from *Eng+/−* mice were stimulated with TGFβ nor did inhibition of the ALK5 kinase by stimulating the cells with SB. Interestingly, when LDN was added to TGFβ stimulated *Eng+/−* macrophage cultures, the differentiation towards M1 macrophages was reduced, resulting in a normalization of the ratio of *Eng+/−* M1–M2 macrophages to WT levels (Figure 3C).

### 2.4. TGFβ/BMP and Non-Smad Signaling in Eng+/− Macrophages Is Impaired

TGFβ transduces its signal from the membrane to the nucleus by phosphorylation of downstream effectors—canonical Smads and non-canonical signaling proteins Erk and p38. To explore which pathway was used, monocytes from WT and *Eng+/−* mice were differentiated into macrophages, serum starved, and stimulated for 60 min with TGFβ, in the absence or presence of the indicated inhibitors SB or LDN. TGFβ was not able to detectably phosphorylate SMAD1/5 after serum starvation in either WT or *Eng+/−* macrophages (data not shown).

**Figure 4:**
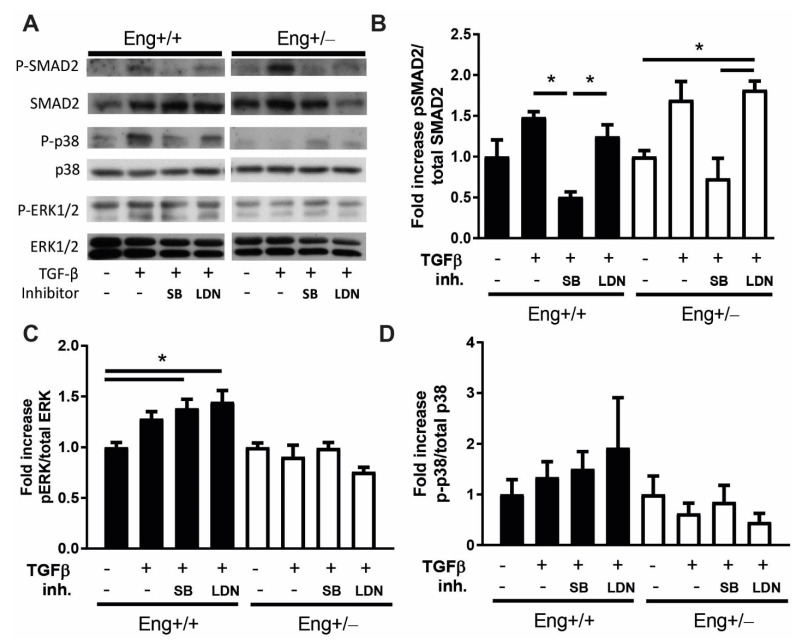
*Eng+/−* macrophages show blunted TGFβ and BMP signaling responses in vitro. (**A**) Western blot analysis of *Eng+/+* and *Eng+/−* cultured murine macrophages with GM-CSF, stimulated 60 min with TGFβ (2.5 ng/uL), SB (10 μM), and LDN (100 nM). Representative blots of N = 3 are shown. (**B**) Densitometric analysis of the blots shown in (A), expressed as percentage of phosphorylated Smad2 relative to total amount of Smad2. N = 3. (**C**) Quantification of the blots as shown in (A), expressed as percentage of phosphorylated ERK relative to total amount of ERK protein. N = 3. (**D**) Quantification of the blots in (A), expressed as the percentage of phosphorylated p38 relative to total amount of p38 protein, N = 3–4. Error bars are SEM. * *p* < 0.05.

Both WT and *Eng+/−* macrophages showed an induction of SMAD2 phosphorylation upon stimulation with TGFβ, which was blocked when SB was added, but not when treated with LDN (Figure 4A,B). Phosphorylation of SMAD2 was not different between WT and *Eng+/−* macrophages. Interestingly, while LDN did not influence TGFβ-induced p-SMAD2 in WT macrophages, a significant increase in the phosphorylation of SMAD2 was observed in the *Eng+/−* macrophages (Figure 4B).

Since TGFβ can also signal via Smad independent pathways [29,30,31], next, we analyzed the non-canonical pathways known to be involved in stress, inflammation, and differentiation responses—the MAPK and p38 pathways. ERK1/2 phosphorylation was increased in WT macrophages upon stimulation with TGFβ, and was further enhanced when SB or LDN were added to TGFβ-stimulated WT macrophages (Figure 4A,C). Macrophages derived from *Eng+/−* mice did not show a change in ERK1/2 phosphorylation when stimulated with TGFβ, in the presence or absence of SB or LDN (Figure 4C). Phosphorylation of p38 showed the same trend as ERK; an increase in p-p38 in WT cells upon TGFβ stimulation and further enhancement in the presence of SB or LDN, while there was no response or even a trend towards reduced p-p38 in *Eng+/−* macrophages (Figure 4A,D). In summary, *Eng+/−* macrophages showed an increase in SMAD2 phosphorylation when BMP signaling was inhibited, while the non-canonical pathways show a decreased responsiveness.

### 2.5. LDN Treatment Improves Cardiac Function after Experimentally Induced MI

**Figure 5:**
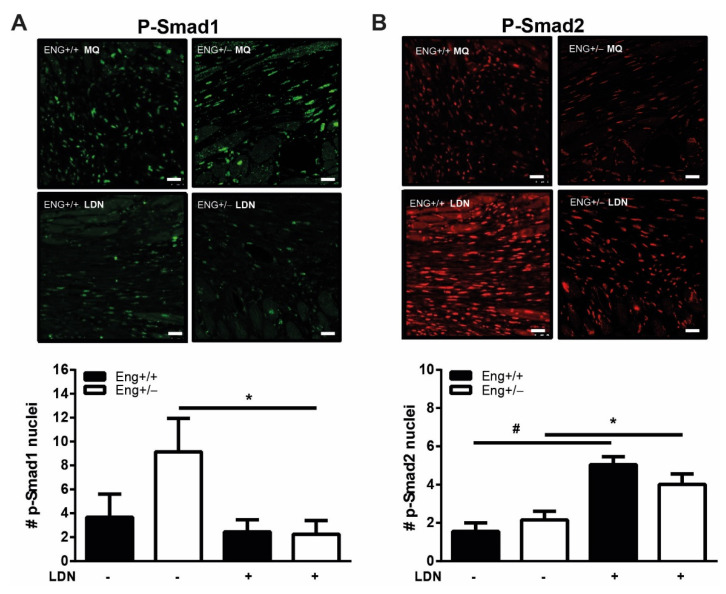
LDN decreases p-Smad1 and induces p-Smad2 in the infarct border zone. Paraffin sections were stained for (**A**) pSmad1 or (**C**) pSmad2 and quantified in (**B**,**D**) for positive stained nuclei in *Eng+/+* and *Eng+/−* mice treated with LDN or placebo. Representative images of heart sections 14 days post-MI are shown. N = 5–16 mice per group. *Eng+/+* control N = 9, *Eng+/−* control N = 5, *Eng+/+* LDN N = 6, *Eng+/−* LDN N = 16. Scale bars: 30 μm. * *p* < 0.05; # *p* < 0.001.

Since LDN influences the TGFβ response in *Eng+/−* macrophages in vitro, we next investigated whether LDN might influence the impaired cardiac recovery of *Eng+/−* mice after MI. LDN was systemically administered 2–14 days after the induction of MI. The efficacy of the LDN treatment was confirmed by a reduction in the number of cells positive for phosphorylated SMAD1 (Figure 5A) and an increased number of cells expressing phosphorylated SMAD2 (Figure 5B), 14 days post-MI.

**Figure 6:**
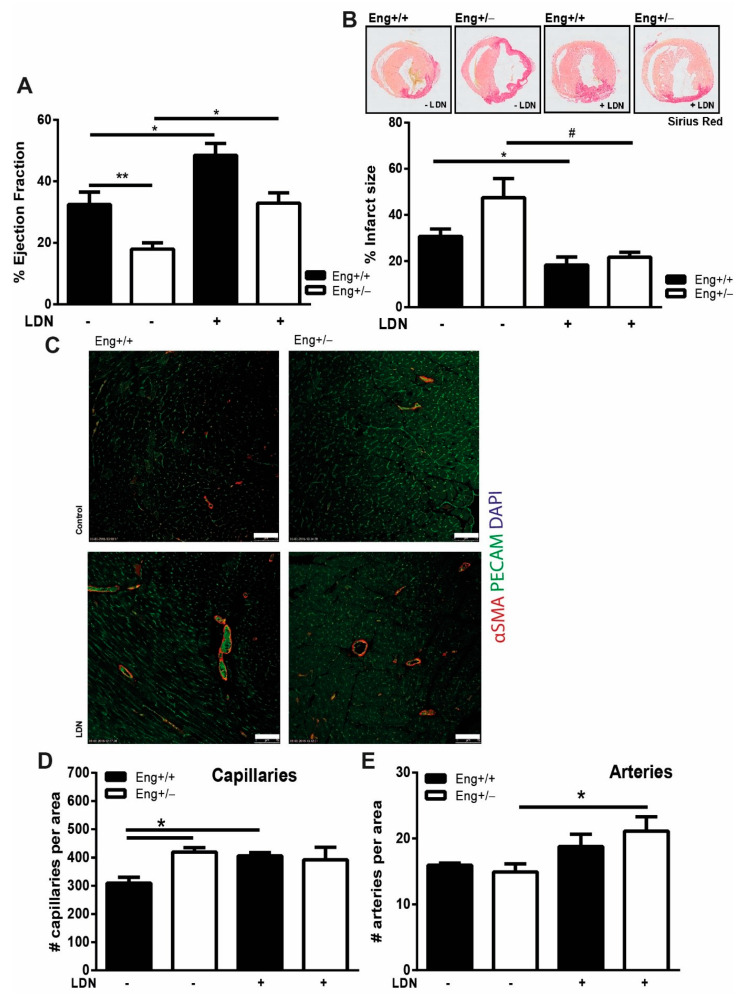
LDN restores cardiac function in *Eng+/−* to normal levels 14 days after MI. (**A**) Cardiac ejection fraction of *Eng+/+* and *Eng+/−* mice 14 days post-MI, treated with LDN or placebo. N = 5–16 mice. (**B**) Infarct size was determined in both *Eng+/+* and *Eng+/−* mice using Picrosirius Red staining. Top row—representative pictures of murine transversal heart sections. 1.0× magnification. Bottom row—quantification of infarcted area as percentage of total LV area. N = 5–16 mice per group. (**C**,**D**) LDN treatment influences cardiac neo-vascularization post-MI. (**C**) Paraffin sections of mouse hearts were stained for PECAM (green) and αSMA (red). N = 5–16 mice per group. *Eng+/+* control N = 9, *Eng+/−* control N = 5, *Eng+/+* LDN N = 6, *Eng+/−* LDN N = 16. (**D**,**E**) Quantification of the number of capillaries (**D**) and arteries (**E**) in (**C**). Scale bar: 50 μm. * *p* < 0.05; ** *p* < 0.01; # *p* < 0.001.

In both WT and *Eng+/−* mice, LDN treatment significantly improved cardiac function (Figure 6A) and reduced infarct size (Figure 6B). Investigating the infarct border zone of these animals in more detail revealed that LDN treatment increased capillary density in WT hearts, but had no effect on the number of capillaries in *Eng+/−* mice. Interestingly, LDN treatment did not change the number of arteries in WT hearts, whereas in *Eng+/−* animals, the number of arteries increased significantly (Figure 6C–E).

### 2.6. LDN Treatment Improves Perfusion Recovery after Hind Limb Ischemia

**Figure 7:**
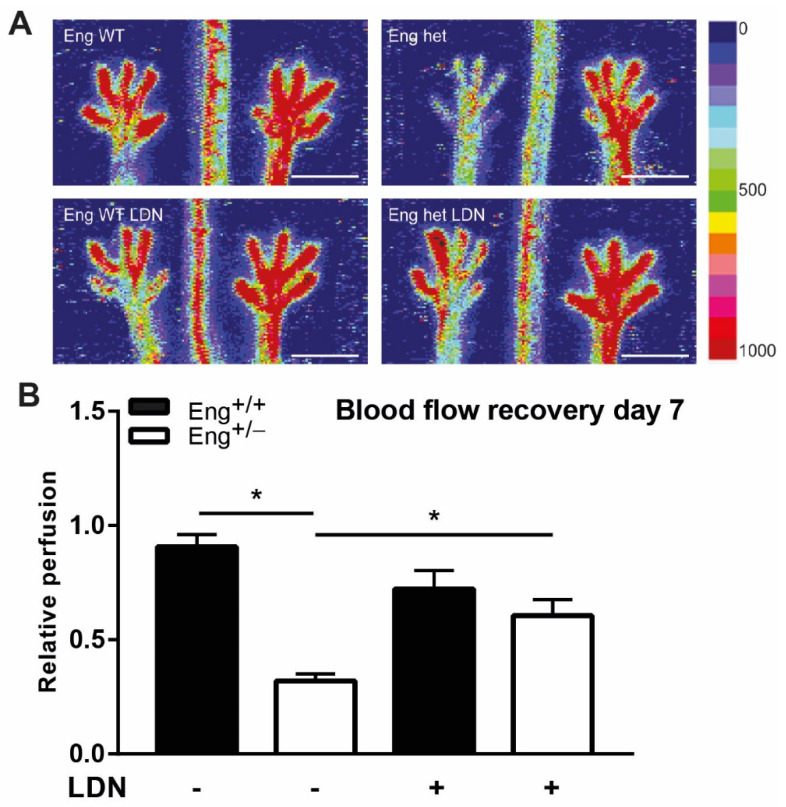
Hind limb blood flow recovery in female mice increases with LDN treatment. (**A**) Representative images of blood flow recovery in the paws as measured by laser Doppler perfusion imaging (LDPI), 7 days after HLI and subsequent treatment with LDN. Colors indicate the level of flow as indicated on the right panel of the figure. The left limb has HLI, the right limb was used as control. Scale bar: 1 cm (**B**) Quantification of LDPI measurements, N = 5–7 female mice per group. Black bars = WT, white bars = *Eng+/−*. * *p* < 0.05.

*Eng+/−* mice show a delayed perfusion recovery after induction of ischemia in the mouse hind limb (Figure 7) [4]. Therefore, to determine whether the effect of LDN was specific for the heart or a more general response of endoglin heterozygosity to an ischemic insult, we chose the hind limb ischemia model in addition to the experimentally induced MI. After ligation of the femoral artery, *Eng+/−* or WT mice were treated with LDN or vehicle, and perfusion recovery was measured by Laser Doppler Perfusion Imaging (LDPI) at day 7 post ligation. Interestingly, while blood flow in the hind limb of WT mice was not different between LDN or vehicle-treated animal, LDN treatment significantly improved the hampered paw perfusion in *Eng+/−* mice (Figure 7). Overall, we conclude that tissue repair in *Eng+/−* mice after ischemic damage in both experimentally induced myocardial and hind limb ischemia was improved by LDN treatment.

## 3. Discussion

The natural response of the body to ischemic injury is to stimulate neovascularization. The influx of circulating monocytes is important for cardiac repair post MI and contributes to the revascularization of ischemic tissue [32,33]. The pro-angiogenic role of endoglin, a TGFβ co-receptor, in vascular development is well established [25,34,35]. We previously reported that the enhanced deterioration of cardiac function after experimentally induced MI in *Eng+/−* mice, results from impaired capacity of HHT1 MNCs to home to the site of injury and accumulate in the infarct zone to stimulate vessel formation [5,36]. In this study, we showed that monocytes depend on the expression of endoglin to be able to differentiate from an inflammatory M1 macrophage towards a reparative M2 macrophage and that *endoglin* heterozygosity prolongs the inflammatory response after myocardial infarction. This observation might explain why patients [1,37,38] and mice [3,11] haplo-insufficient for *endoglin* show prolonged inflammation and delayed wound healing and tissue repair after injury.

We demonstrated that TGFβ differently influenced the differentiation of wild type versus *Eng+/−* or HHT1 macrophages. Wild type monocytes differentiate to M2 macrophages in an ALK5-dependent manner, while inhibition of the BMP type I receptors did not influence their differentiation. Macrophages heterozygous for *endoglin* did not differentiate towards M2 upon TGFβ stimulation, while inhibition of BMP-signaling resulted in a shift towards M2 macrophages. TGFβ is well-known as an anti-inflammatory/pro-fibrotic cytokine and is mainly secreted by M2 macrophages [39,40]. We previously showed that there was no difference in TGFβ, TβRII, ALK1, and ALK5 expression in WT vs. HHT1 mononuclear cells [5], and endothelial cells deprived of endoglin expression were unable to process and secrete active TGFβ [41]. We hypothesized that the defect in TGFβ/BMP ligand processing due to deficiency in the co-receptor endoglin could play a role in the impaired TGFβ-directed differentiation of *Eng+/−* macrophages and explain why inhibition of BMP signaling could restore defective endoglin/TGFβ signaling. The reduced levels of endoglin might skew the tight balance that often exists between TGFβ and BMP signaling, and inhibition of the BMP type I receptor kinase might push this balance towards enhanced TGFβ signaling, thereby restoring the balance and M2 macrophage differentiation.

The main defect observed in TGFβ signaling in *Eng+/−* monocytes was related to the non-Smad signaling pathway. *Eng+/−* macrophages were still able to signal via the canonical TGFβ/SMAD pathway and phosphorylation of Smad2 was significantly increased when TGFβ was present in combination with LDN. Endoglin has two splice isoforms, a long form (L-Endoglin) and a short form (s-endoglin), containing a 33 amino acid shorter cytoplasmic tail [42]. Both isoforms are able to bind TGFβ but differ in phosphorylation status and their ability to interact with ALK1 and ALK5 [43,44]. A recent study showed that hypoxia-induced expression of S-endoglin stimulates signaling via TGFβ/ALK5/Smad2, causing impaired angiogenesis in the pulmonary vasculature of the developing lung [45]. Although we analyzed the impact of *endoglin* heterozygosity, determining which isoform of endoglin is involved in the pathology of HHT is an interesting topic for future research.

We observed reduced activation of the non-canonical MAPK/ERK pathway. An overall imbalance in the ERK and p38 signaling has a pronounced effect on the inflammation status and in reaction to stress [46]. Previous studies reported the involvement of endoglin in the MAPK/ERK pathway. In dermal fibroblasts, *endoglin* haploinsufficiency did not affect the basal or TGFβ induced pERK1/2, while the basal levels of Akt show a higher degree of phosphorylation [47]. In endothelial cells, the activation levels of Akt was not different between WT and *Eng+/−* cells, while ERK and p38 signaling was more active in *Eng+/−* endothelial cells [48]. We showed that in macrophages heterozygous for *endoglin*, ERK1/2 phosphorylation was impaired and neither stimulation nor inhibition of TGFβ signaling resulted in the phosphorylation of ERK1/2. ERK signaling is involved in cell growth and differentiation [49], and might affect apoptosis [50]. Defects in these aforementioned processes could explain the prolonged inflammatory status we observed in *Eng+/−* mice. In addition to impaired ERK activation, *Eng+/−* macrophages showed decreased phosphorylation of p38 in response to TGFβ stimulation and BMP inhibition. P38 is involved in TGFβ-directed monocyte migration and inhibits monocyte proliferation [51], has anti-angiogenic properties, and is reported to be involved in maintaining a proper balance in the angiogenic response [52]. Phosphorylated p38 was reported to inhibit VEGF signaling [53], and *Eng+/−* cells exhibit increased VEGF expression [48]. Therefore, the reduced levels of p-p38 could explain the endothelial hyperplasia and impaired angiogenesis found in HHT1 patients [54,55].

In the present study we showed that the inability of MNCs from HHT1 patients to induce neoangiogenesis post MI was not solely due to an impaired recruitment of the MNCs to the site of injury [36], but was also a result of impaired macrophage differentiation, mainly towards an inflammatory phenotype, which would intervene with myocardial repair [56]. High levels of inflammatory macrophages correlate with ventricular dysfunction after MI, in both mice and patients [57,58]. Selective depletion of M2 macrophages resulted in a nine-fold increase in cardiac rupture [59], and interfered with differentiation into M2 macrophages by knocking out tribbles pseudokinase 1 impaired ventricular function, 7 days post MI [60]. These studies, support our observation that M2 macrophages show a beneficial role in tissue remodeling. The impaired differentiation towards the reparative macrophage subtype due to *endoglin* heterozygosity could be restored by inhibiting the BMP type I receptor kinase with the small molecule inhibitor LDN, confirming both a BMP-dependent and non-canonical modulation of macrophage function in HHT1. Furthermore, cardiac ejection fraction after MI and reperfusion recovery after HLI were improved when *endoglin* heterozygous mice were treated with LDN. An important limitation of our study is that we used a non-reperfused myocardial infarction model, causing the infarct zone to be cut off from the circulation. The main effect of our LDN inhibitor is therefore likely of greatest impact at the infarct border zone, where blood vessels are still intact and tissue is perfused. Future research should include ischemia-reperfusion models to assess the effect of LDN on cardiac repair. Cumulatively, our results imply that treating HHT1 patients with a BMP type I receptor kinase inhibitor would improve tissue repair, and could be considered as a novel therapeutic target in patients with ischemic tissue damage.

## 4. Materials and Methods

### 4.1. Clinical Studies

The procedures performed were approved by the Medical Ethics Committee of the St. Antonius Hospital Nieuwegein, The Netherlands. The study conformed to the principles outlined in the 1964 Declaration of Helsinki and its later amendments. All persons gave their informed consent prior to their inclusion in this study. Venous blood samples from 7 HHT1 patients and 5 age- and gender-matched healthy human volunteers were collected. Peripheral blood MNCs were isolated by density gradient centrifugation using Ficoll Paque Plus (GE Life sciences, Zwijndrecht, The Netherlands, #17-1440-02), according to the manufacturer’s protocol.

### 4.2. Animals

All mouse experiments were approved by the regulatory authorities of Leiden University, The Netherlands (ADEC nr 14-141) and were in compliance with the guidelines from Directive 2010/63/EU of the European Parliament, on the protection of animals used for scientific purposes, approved date 16 September 2014. Experiments were conducted in 10–12 weeks old *Eng+/+* and *Eng+/−* male or female C57BL/6Jico mice (Charles River, Leiden, The Netherlands).

### 4.3. Myocardial Infarction and BMPRI-Inhibitor Treatment

Myocardial infarction (MI) was induced in male mice, as described before [36]. Briefly, mice were anesthetized with isoflurane (1.5–2.5%), intubated and ventilated, after which the left anterior descending coronary artery was permanently ligated. The mice were given the analgesic drug Temgesic, both pre-operative and 24 h post-operative to relieve pain. Mice were randomly allocated to the treatment or placebo control groups. Placebo or BMPRI-inhibitor [61] LDN-193189 was reported to inhibit the kinase activity of the BMP type I receptors ALK1/2 (IC50 = 5 nM) [62], leaving the TGFβ/ALK5 pathway unaffected. LDN-193189 (2.0 mg/kg, Axon Medchem, Groningen, The Netherlands, #Axon1509) was administered twice daily via intraperitoneal injection from 2 days after MI till day 14. Heart function was measured by echocardiography 14 days post-MI, after which the hearts were isolated and fixated in 4% paraformaldehyde (in PBS) and embedded in paraffin.

Mice were monitored daily by the researchers or animal care staff to check their health and behavior for human endpoints. All were trained in animal care and handling and determining the following criteria and symptoms—impaired reaction to external stimuli, reduced mobility, or decreased grooming. Furthermore, for 3 days post-MI and onwards—bleeding, swelling, redness, or discharge of the incision area. Mice were weighed at day of surgery and prior to echocardiography and euthanized by carbon dioxide, when losing more than 15% weight. Animals dropped out prior just after MI or within 10 days post-MI- due to cardiac rupture.

### 4.4. Cardiac Function Measurements

Echocardiography was performed after mice were anesthetized with 1.5–2.5% isoflurane using the Vevo 770 (VisualSonics, Inc., Toronto, ON, Canada) system, with a 30 MHz transducer (RMV707B). We imaged the longitudinal axis of the left ventricle using the Electrocardiography-based Kilohertz Visualization (EKV) imaging mode. The ejection fraction was determined by tracing the left ventricular volume during the systolic and diastolic phase, using the Vevo770 V3.0 imaging software (VisualSonics, Inc., Toronto, ON, Canada).

### 4.5. Hind Limb Ischemia and Perfusion Imaging

Hind limb ischemia (HLI) was induced as described before [63]. In brief, male and female mice were anesthetized by intraperitoneal injection of midazolam (8.0 mg/kg, Roche Diagnostics, Almere, The Netherlands), medetomidine (0.4 mg/kg, Orion, Espoo, Finland), and fentanyl (0.08 mg/kg, Janssen Pharmaceuticals, Beerse, Belgium). Ischemia of the left hind limb was induced by electrocoagulation of the left femoral artery, the right hind limb served as control. After surgery, anesthesia was antagonized with flumazenil (0.7 mg/kg, Fresenius Kabi), atipamezole (3.3 mg/kg, Orion), and buprenorphine (0.2 mg/kg, MSD Animal Health, Boxmeer, The Netherlands).

Blood flow recovery to the hind limb was measured using laser Doppler perfusion imaging (LDPI, Moore Instruments, Axminster, UK), at 7 days post injury. During LDPI measurements, mice were anesthetized by intraperitoneal injection of midazolam (8.0 mg/kg, Roche Diagnostics) and medetomidine (0.4 mg/kg, Orion). After LDPI, anesthesia was antagonized by subcutaneous injection of flumazenil (0.7 mg/kg, Fresenius Kabi, Bad Homburg vor der Höhe, Germany) and atipamezole (3.3 mg/kg, Orion). Humane endpoints after induction of HLI were considered when mice were less mobile, showed impaired reaction to external stimuli, or decreased grooming. Furthermore, when the incision wound area was bleeding, swollen, or discharged, animals would be euthanized by carbon dioxide.

### 4.6. Immunohistochemistry

After euthanasia by carbon dioxide, mouse hearts were dissected, fixated overnight in 4% paraformaldehyde (in PBS) at 4 °C, dehydrated, and embedded in paraffin wax. Six μm sections were baked onto coated glass slides (VWR, Amsterdam, The Netherlands, SuperFrost Plus), and stained for the presence of macrophages in the infarct border zone, using rat anti-mouse MAC3 (CD107b, dilution 1:200, BD Biosciences, San Jose, CA, USA, #550292) and goat anti-rat biotinylated secondary antibody (1:300, Vector Laboratories, Burlingame, CA, USA, #BA-9400). An avidin/biotin-based DAB peroxidase staining was used (Vectastain ABC system, Vector Laboratories, #PK-4000) to detect antibody binding, next to a hematoxylin counterstain for cell nuclei.

Infarct size was determined by Picrosirius Red (PSR) collagen staining; slides were deparaffinized and hydrated, followed by 1 h incubation with PSR solution; 0.1gram Sirius Red F3B (Merck, Zwijndrecht, The Netherlands) dissolved in 100 mL saturated picric acid solution (pH = 2.0) (Sigma, Zwijndrecht, The Netherlands, #P6744). Slides were washed in acidified water, dehydrated in ethanol and mounted with Entellan (Merck) mountant.

Immunofluorescent stainings were performed using standard protocol as previously described [64] for visualization of capillaries by PECAM (CD31, dilution 1:800, Santa Cruz, #sc-1506), arteries by both PECAM (CD31, dilution 1:800, Santa Cruz Biotechnology Inc. Heidelberg, Germany) and αSMA (alpha smooth muscle actin, dilution 1:500, Abcam). Macrophages were stained with CD11b (MAC-1, dilution 1:200, Biolegend, London, UK #1012505, clone M1/70), MAC-3 (CD107b, dilution 1:200, BD Biosciences, #550292), and CD206 (dilution 1:300, Abcam, #ab64693). Simultaneously, detection of p-Smad1/5/8 (dilution 1:100, Cell signaling, Bioke, Leiden The Netherlands, #9511) was performed by 30 min antigen retrieval and subsequently p-Smad2 (dilution 1:200, Cell signaling, #3101) was amplified using a TSATM-Biotin System (Tyramide Signal Amplification) Kit (Perkin Elmer Life Science, Waltham Massachusetss, USA, #NEL700A). Fluorescent-labelled secondary antibodies (ThermoFisher Scientific, Bleiswijk, The Netherlands) were incubated for 1.5 h, at 1:250 dilutions. Sections were mounted with Prolong^®^ Gold Antifade mountant with DAPI (ThermoFisher Scientific, # P36931).

### 4.7. Isolation of Immune Cells from Murine Hearts

Hearts were harvested from the mice 4 days post- MI and put in PBS buffer on ice. After excision of the left ventricle, the tissue was put into 1-mL digestion buffer (450U Collagenase A, Sigma Aldrich, Zwijndrecht, The Netherlands, # 10103578001, 60U hyaluronidase, Sigma Aldrich, #H3506, 60U DNAse-1, Roche, #10104159001) at 37 °C for 1 h. The tissue homogenate was filtered through an 80-μm cell strainer (Falcon # 352350) and MNCs were isolated using Ficoll density gradient, specific for small mammalians (Histopaque-1083, Sigma Aldrich # 10831). Flow cytometry and staining was performed as described below.

### 4.8. Flow Cytometry

Mouse monocytes from either 50 μL of whole blood, bone marrow mononuclear cells, or from heart lysates were stained for CD11b (with anti-mouse CD11b, BD Biosciences, #561114), Ly6C (Bio-rad Laboratories, Veenendaal, The Netherlands # MCA2389A647T or BD Biosciences, #561085), and CD206 (Bio-rad Laboratories, #MCA2235A488T), to identify M1 and M2 macrophages, respectively.

Human monocytes were isolated from peripheral blood, through Ficoll gradient separation. Total MNCs (3× 105 cells per sample) were stained with anti-CD14-ECD (Beckman Coulter, Woerden, The Netherlands IM2707U) and anti-CD16-APC (Beckman Coulter, # A66330). Fluorescence was measured with LSRII flow cytometer (BD Biosciences) and analyzed by FACS Diva (BD Biosciences) and the FlowJo software (FlowJo LLC V9.4).

### 4.9. Cultured Macrophages from Mouse Bone Marrow

Monocytes were isolated from the bone marrow using CD11b+ magnetic beads (Miltenyi Biotec MACS #130-049-601) and subsequently cultured in the RPMI medium (Gibco RPMI 1640 Medium, #11875-093), supplemented with 10% FBS (Fetal Bovine Serum, Gibco, ThermoFisher Scientific, #10270), and 1 ng/mL GM-CSF (Peprotech, #315-03), to induce differentiation into macrophages. After 3 days, the attached cells were stimulated with TGFβ3 (1 ng/mL, kind gift of Dr. K. Iwata), ALK5 kinase was inhibited using SB-431542 (10 μM, Tocris, Abingdon, UK #1614), and BMPR type I (ALK1/2/3) were inhibited using LDN-193189 (100 nM, Axon Medchem, #Axon1509) addition for 4 days.

### 4.10. Western Blot Analysis

For intracellular protein analysis, at day 6 of culture, the macrophages were serum starved overnight, after which they were either stimulated or not for 1 h with TGFβ or inhibitors at the indicated concentrations, after which cells were lysed with RIPA lysis buffer (5 M NaCl, 0.5 M EDTA, pH 8.0, 1 M Tris, pH 8.0, NP-40 (IGEPAL CA-630), 10% sodium deoxycholate, 10% SDS, in dH2O) supplemented with phosphatase inhibitors (1M NaF Sigma Aldrich # S7920, 10% NaPi Avantor #3850-01, 0.1M NaVan Sigma Aldrich # S6508) and protease inhibitors (complete protease inhibitor cocktail tablets, Roche Diagnostics, #11697498001). Protein concentration was measured using Pierce BCA protein assay (ThermoFisher Scientific, #23225). Equal amounts of protein were loaded onto 10% SDS-polyacrylamide gel and transferred to an Immobilon-P transfer membrane (PVDF membrane, Millipore, # IPVH00010). The blots were blocked for 1 h using 5% milk in TBST (Tris-buffered saline, 0.1% Tween20) solution and incubated O/N with rabbit anti-mouse phosphorylated Smad2 (Cell signaling, #3101), total Smad2/3 (BD Biosciences, BD610842), mouse anti-mouse phosphorylated ERK1/2 (Sigma-Aldrich, #M8159), rabbit anti-mouse total ERK1/2 (p44/42 MAPK, Cell Signaling, #4695, clone 137F5), rabbit anti-mouse phosphorylated p38 (Cell Signaling Technology, #9211), and mouse anti-rabbit total p38 (Santa Cruz Biotechnology Inc. #535). Blots were incubated for 60 min with horse radish peroxidase anti-rabbit (ECL rabbit IgG, HRP-linked whole Ab, GE Healthcare, #NA934V) or anti-mouse (ECL mouse IgG, HRP-linked whole Ab, GE Healthcare, #NA931V) antibodies. Blots were developed in an X-omat 1000 processor (Kodak) with SuperSignal West Dura Extended Duration Substrate (ThermoFisher Scientific, #37071) or SuperSignal West Pico Chemiluminescent Substrate (ThermoFisher Scientific, # 34080AB), and exposed to SuperRX medical X-ray film (Fujifilm Corporation). Analysis was performed using Image J (Version 1.51, https://imagej.nih.gov/ij/index.html (accessed on 17 February 2021), National Institutes of Health, Bethesda, MD, USA).

### 4.11. Morphometry

Tissues were sectioned using a cryotome (hind limb muscle) or microtome (cardiac tissue) at approximately 300 µm intervals along the ischemic area. Cardiac infarct size was determined using Picrosirius Red staining and calculating the percentage of the infarct area to the total area of the left ventricle. Cell infiltration was determined by manual quantification of 2 to 4 digital images per heart at the border zone facing the infarcted area or hind limb, taken at 40× magnification (CaseViewer 3D Histech). The quantification of the number of capillaries and arteries present was done manually in the border zone surrounding the ischemic area, where (cardio)myocytes were still viable. Data were blinded to the investigator and quantified by using ImageJ v1.46r (https://imagej.nih.gov/ij/index.html (accessed on 17 February 2021), National Institutes of Health, Bethesda, MD, USA).

### 4.12. Statistical Analysis

Statistical significance was evaluated by the D’Agostino-Pearson normality test, followed by Mann-Whitney (non-parametric) or unpaired Student’s t-test (parametric) between 2 groups. To perform testing between multiple groups, ANOVA (parametric) with Bonferroni correction or Kruskal-Wallis (non-parametric) test was used. Analysis was performed with GraphPad Prism 6 software. Values are represented as mean ± SD or SEM when otherwise indicated. Values of *p* < 0.05 are denoted as statistically significant.

### 4.13. Data Availability

No datasets were generated or analyzed during the current study. The results generated during or analyzed during the current study are available from the corresponding author on reasonable request.

## Data Availability

The data presented in this study are available on request from the corresponding author.

## Supplementary Materials

The following are available online at https://www.mdpi.com/1422-0067/22/4/2010/s1, Figure S1: *Endoglin* heterozygous macrophages secrete more MCP-1 compared to wild type cells. MNC cells were cultured for 24 h after which the amount of MCP-1 present in the medium was determined using an ELISA assay.
