# Supplementary material for: BMP Receptor Inhibition Enhances Tissue Repair in Endoglin Heterozygous Mice
Source: Int J Mol Sci. 2021 Feb 18;22(4):2010. doi: 10.3390/ijms22042010 (PMC7922601; doi:10.3390/ijms22042010)
Supplement: Supplementary file 1 [file ijms-22-02010-s001.pdf]

Supplementary data

Supplementary figure 1

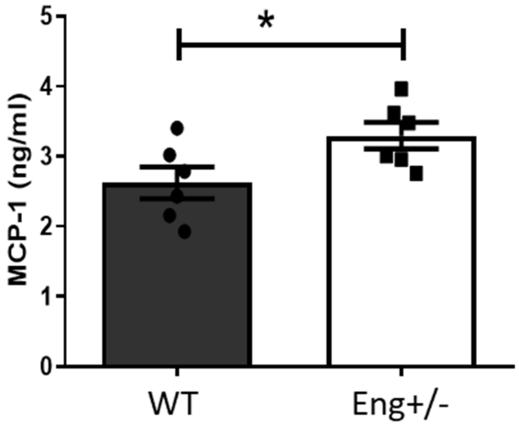

Figure S1: Endoglin heterozygous macrophages secrete more MCP-1 compared to wild type cells. MNC cells were cultured for 24 hours after which the amount of MCP-1 present in the medium was determined using an ELISA assay. ELISA for MCP-1 on cell-free supernatants was carried out with the MCP-1 kit from Antigenix (catalog#RRF423CK) following the manufacturer's instructions. * $p < 0.05$.
